# What students ask matters: Static and longitudinal questioning patterns in student–LLM dialogue and self-reported cognitive engagement

**DOI:** 10.1371/journal.pone.0356183

**Published:** 2026-09-08

**Authors:** Jingru Qu, Ling Dai, Mengjie Yin

**Affiliations:** 1 School of Humanities and Social Science, the Chinese University of Hong Kong, Shenzhen, China; 2 Institute of Education, Nanjing University, Nanjing, China; University of Oxford, UNITED KINGDOM OF GREAT BRITAIN AND NORTHERN IRELAND

## Abstract

This study examines students’ questioning as an interaction mechanism in student–LLM philosophical dialogue and how it relates to self-reported critical thinking (CT) and creative thinking (CrT) engagement. Participants were 106 first-year postgraduate students in a Chinese university general education course who completed an end-of-semester assignment using an institutional ChatGPT model embedded in the learning platform. Student questions in dialogue transcripts were coded into six categories aligned with the revised Bloom’s taxonomy. We then applied latent profile analysis (LPA) to identify static questioning profiles and group-based trajectory modeling (GBTM) to capture longitudinal development across conversation rounds, followed by subgroup comparisons on perceived CT and CrT. LPA yielded three profiles: Fact-focused, Explanation-focused, and Evaluation-focused Questioners. GBTM revealed two trajectories with a key divergence around Rounds 5–7: some students plateaued at application and analytical questioning, while others progressed toward evaluative and exploratory questioning. Fact-focused Questioners reported lower CT and CrT engagement scores; plateauing trajectories reported lower CT. Findings highlight how questioning patterns are associated with differential self-reported cognitive engagement in student–LLM dialogue and inform scaffold design to promote higher-order inquiry.

## 1. Introduction

As conversational AI, particularly in the form of LLMs, is increasingly integrated into higher education through chat-based tutoring, feedback, and dialogue tasks, its potential influence on students’ thinking has attracted growing attention [1–5]. Beyond academic performance and writing quality, a central concern is whether student interaction with LLM can foster higher-order capacities such as critical thinking (CT) and creative thinking (CrT)—competencies that are foundational to university learning and to engaging with complex, ill-structured problems [6,7].

At the same time, the field is moving beyond a binary debate about whether LLM use is beneficial or harmful for thinking. Recent scholarship reflects a constructivist, process-oriented turn that emphasizes how students use LLMs, how interaction unfolds, and which interaction patterns are more conducive to cognitive development and knowledge construction [2,4,8–12]. This shift resonates with longstanding concerns that learning outcomes depend on how learners are positioned within activity structures and dialogue, whether they are primarily responding to externally imposed prompts or actively initiating inquiry to advance understanding [13–16]. LLMs make this issue especially salient because system outputs are contingent on learner inputs, rendering interaction patterns a plausible mechanism linking LLM use to cognitive outcomes.

Longstanding scholarship suggests both the promise and fragility of questioning for learning. In philosophical work on critical thinking, questioning is framed as an epistemic process rooted in fallibilism, the recognition that beliefs are revisable and must be evaluated and justified through inquiry [17–21]. Yet students may also approach learning as locating a correct answer and terminating inquiry once an answer is obtained, which can limit deeper exploration of assumptions, implications, and alternatives [17]. Classroom research likewise documents how teacher-dominated discourse patterns can suppress authentic student questioning [14–16], while more dialogic and less threatening arrangements can make student-generated questions more likely to emerge [13]. Together, these perspectives motivate closer examination of students’ questioning in student–LLM dialogue as a process through which cognitive engagement and thinking outcomes may be shaped.

Within student–LLM dialogue, interaction is frequently carried by questioning: students advance the conversation by requesting information, seeking clarification, probing assumptions, or soliciting alternative viewpoints. Questioning therefore functions not only as a discourse feature but as an epistemic mechanism that can keep inquiry open and generative or close it around answer-getting. In philosophical accounts of critical thinking grounded in fallibilism, questioning is central to evaluating and justifying beliefs, and different kinds of questions support qualitatively different levels of cognitive engagement [17–21]. As [17] states, “With respect to the primary and secondary functions of questioning, we may distinguish between fact-finding questions and analytical questions” (pp. 335–336). Complementing this epistemic framing, empirical work in inquiry contexts characterizes student questioning as a dynamic interaction mechanism that can evolve, transform, and intensify as learners encounter conflicts between observations, prior beliefs, and peer contributions [22,23]. This makes students’ questioning a particularly promising lens for examining how student–LLM dialogue may differentially support higher-order thinking across learners and across the unfolding course of interaction.

In higher education, the integration of LLMs and GenAI is expanding rapidly across disciplines and instructional purposes, yet many implementations still remain at an augmentation level rather than enabling more innovative learning designs [24]. Correspondingly, scholars have argued that students need prompt-related competencies, AI literacy, and ethical understanding to translate access into meaningful learning benefits [25]. Syntheses of GenAI pedagogy similarly emphasize higher-order goals, including CT, CrT, learner autonomy, and prompt literacy [5]. However, students’ everyday use does not consistently reflect these aspirational goals. Large-scale evidence suggests that even when GenAI use is nearly universal, students often concentrate on relatively basic applications such as translation and proofreading, with comparatively limited use for cognitively demanding activities [26]. This mismatch between educational aims and students’ enacted practices highlights the need for research that characterizes what students actually do in student–LLM dialogue and identifies interaction patterns that are more likely to support higher-order learning.

Empirical studies on student–LLM interaction have begun to document promising effects on higher-order learning, particularly for CT. For example, AI-assisted debate and discussion designs have been associated with stronger performance in conceptual understanding, CT, and argumentation compared with non-AI conditions [27], and a growing body of experimental and quasi-experimental research reports gains in higher-order thinking outcomes when LLMs are incorporated into learning activities [2,8,9,28]. At the same time, a mechanism-focused interpretation is increasingly necessary because findings also suggest that benefits are conditional. LLMs can reduce the burden of information gathering and broaden accessible perspectives, potentially allowing learners to allocate more effort to evaluation and reflection [29,30]. Yet the same affordances may also invite cognitive offloading, uncritical acceptance, and dependency when students adopt AI outputs without active processing [3,31–33]. A parallel pattern appears in creativity research: GenAI may diversify ideation and lower barriers to expression in some designs [8,34], but other evidence suggests limited creativity advantages and raises concerns that reliance on standardized AI responses may constrain originality [35]. Taken together, this literature implies that CT and CrT outcomes are not determined by LLM presence per se, but by how students engage during student–LLM dialogue.

## 2. The present study

The present study investigates how students’ questioning functions as a key interaction mechanism in student–LLM philosophical dialogue, and how this mechanism is related to self-reported CT and CrT engagement. Using students’ dialogue transcripts from an authentic end-of-semester course assignment, we analyze what kinds of questions students ask and how their questioning changes as the dialogue unfolds.

The study addresses three research questions:

RQ1: What static questioning profiles emerge in student–LLM philosophical dialogue?
RQ2: What questioning trajectories emerge across conversation rounds?
RQ3: Are these profiles or trajectories associated with differences in self-reported CT and CrT engagement?

This work is significant for educational technology research and practice in three respects. Conceptually, it advances the field’s process-oriented agenda by moving from broad claims about the effects of LLM to a mechanism-focused account of how learning may be shaped through interaction, positioning questioning as a theoretically grounded lever for higher-order thinking [17,22,23,36]. Methodologically, it demonstrates how naturally occurring dialogue data can be used to identify heterogeneous patterns and developmental pathways rather than relying only on average effects. Practically, by identifying questioning patterns associated with differential CT and CrT engagement, it provides an evidence base for designing instructional scaffolds and prompting supports that encourage more cognitively generative student–LLM dialogue in higher education.

## 3. Methods

A sequential explanatory mixed-methods design [37] was employed to comprehensively elucidate students’ questioning mechanisms during student–LLM philosophical dialogue. This design integrates qualitative and quantitative data to address complex educational phenomena [38], with four distinct phases capturing both static and longitudinal dimensions of questioning patterns: qualitative content analysis of chat logs; latent profile analysis (LPA) to identify static subgroups based on questioning type proportions; group-based trajectory modeling (GBTM) to reveal longitudinal trajectory patterns; and statistical comparison of subgroups and trajectory groups on cognitive outcome measures.

### 3.1 Participants and setting

The final analytic sample comprised 106 first-year postgraduate students from a Chinese university, including 49 females and 57 males spanning humanities, social sciences, business, arts, and STEM majors. Participants were drawn from Dialectics of Nature, a required general education course covering the philosophy and history of science and dialectics. As a required end-of-semester assignment, students engaged in a student–LLM dialogue using an institutional ChatGPT model embedded in the university’s learning platform and discussed one of two prompts: “Artificial Intelligence and Technological Ethics” or “The Relationship Between Science and Philosophy.” After completing the dialogue, students submitted their chat logs for grading and completed an online questionnaire. Eligibility for inclusion in the final sample was determined via questionnaire screening to ensure participants had comparable baseline levels of prior GenAI/LLM experience, AI-related knowledge, self-reported familiarity with AI tools, and frequency of AI use, thereby minimizing confounding influences of prior AI exposure on subsequent questioning behaviors.

### 3.2 Measures

#### 3.2.1 Online questionnaire.

*Perceived Creative Thinking (CrT)* was measured with a five-item, six-point Likert scale adapted from the Use of Creative Cognition Scale [39]. A sample item is “While working on this assignment, I try to generate as many ideas about the topic as possible.” The scale demonstrated excellent internal consistency with a Cronbach’s α of 0.901. Exploratory factor analysis (EFA) confirmed its construct validity, with the Kaiser-Meyer-Olkin measure of sampling adequacy at 0.846 and a significant Bartlett’s Test of Sphericity (χ²(10) = 1042.804, *p <* 0.001). Principal component analysis extracted a single robust component with an eigenvalue of 3.587, accounting for 71.74% of the total variance.

*Perceived Critical Thinking (CT)* was measured using a five-item, six-point Likert subscale adapted from the Motivated Strategies for Learning Questionnaire [40]. A sample item is “When completing this assignment, I often find myself questioning the content I refer to (e.g., literature, viewpoints) to decide if I find them convincing.” The scale showed outstanding reliability with a Cronbach’s α of 0.943. Its construct validity was confirmed through EFA, with a KMO measure of 0.872 and a significant Bartlett’s Test of Sphericity (χ²(10) = 1878.442, *p <* 0.001). Principal component analysis extracted a single component accounting for 82.23% of the total variance, indicating excellent construct validity.

#### 3.2.2 Grading rubrics.

To obtain objective performance-based indicators of students’ higher-order thinking demonstrated in the assignment, the submitted student–LLM dialogue logs were evaluated using two analytic rubrics: the Critical Thinking Analytic Rubric [41] and a Creative Thinking Rubric adapted from scoring principles commonly associated with the Torrance Tests of Creative Thinking [42].

Critical Thinking Analytic Rubric (CTAR). The CTAR assesses critical thinking through six dimensions—Identification, Analysis, Evaluation, Inference/Reasoning, Explanation, and Cognition—each rated on a 6-point scale (1 = lowest, 6 = highest). Across dimensions, higher scores reflect more accurate identification and interpretation of key claims and evidence, deeper comparative analysis across viewpoints, stronger evaluation of arguments from multiple perspectives, more warranted inferences and implications, clearer evidence-based explanation supporting conclusions, and more context-sensitive and epistemically open reasoning. Dimension scores were aggregated to yield an overall CT score for each student.

*Creative Thinking Rubric (CTR).* The CTR assessed creative thinking using three TTCT-aligned indicators: Fluency, Flexibility, and Originality [42]. For each student, raters segmented the written product into idea units and applied a 4-level rating (0–3) to each unit for the relevant indicator. Following the scoring scheme, ratings were converted into a 0–12 scale per indicator: 0 = no response (0 points); 1 = low-level performance (1–4 points; e.g., unrelated/repeated ideas for Fluency; no transformation for Flexibility; no originality evidence for Originality); 2 = moderate performance (5–8 points; incomplete idea units/transformations or rare originality); and 3 = high performance (9–12 points; complete idea units/transformations or more frequent originality evidence). The three indicator scores were then aggregated to yield an overall CrT performance score.

### 3.3 Process

#### Phase 1: Qualitative content analysis of questioning patterns.

A deductive coding framework was developed based on the revised Bloom’s Taxonomy [10,43], with the unit of analysis defined as each distinct question posed by students. Six mutually exclusive categories were established to capture the full spectrum of cognitive engagement. Factual questions focus on retrieving specific facts, definitions, terminology, or basic concepts. Explanatory questions aim to clarify ideas, concepts, or processes in one’s own words. Application questions center on using information in new or concrete situations, such as solving problems or applying theoretical concepts to practical examples. Analytical questions involve breaking down information into constituent parts to explore relationships, identify patterns, or distinguish between components. Evaluative questions require making judgment calls about the value of ideas, solutions, or materials based on specific criteria and standards. Exploratory questions target generating new ideas, products, or perspectives by reorganizing elements into new patterns or structures.

To illustrate the operational boundaries of these categories, the following anonymized examples are drawn from the corpus: Factual—“What is the definition of the simulation hypothesis? ”; Explanatory—“Could you explain in more detail how Kuhn’s paradigm shifts work? ”; Application—“If technology replaces human judgment, how would that apply to medical diagnosis?”; Analytical—“What are the differences between the deontological and utilitarian perspectives on this issue? ”; Evaluative—“Do you think this argument is convincing? What are its limitations?”; Exploratory—“Can we imagine an alternative framework for understanding the relationship between AI and human creativity? ”

The complete corpus of 106 chat logs was imported into MAXQDA 2020 for systematic analysis. Two independent coders, postgraduate students in educational technology, were trained on a detailed coding manual specifying category definitions, boundary rules, and exemplar questions for each of the six categories. The coders first double-coded a randomly selected 20% of the data (308 of 1,538 questions). Inter-rater reliability was assessed using Cohen’s κ, yielding κ = 0.704, indicating substantial agreement [44]; simple percentage agreement was 76.3%. Disagreements were discussed and resolved through consensus, with the first author serving as a third senior researcher. Following this calibration phase, which established a shared understanding of the coding criteria, one coder completed the remaining 80% of the dataset. Throughout this phase, the coder consulted the coding manual for boundary cases and discussed uncertain items with the first author before final assignment.

#### Phase 2: LPA– Static questioning patterns.

LPA was employed to identify naturally occurring subgroups of students based on their distinct questioning patterns [45]. As a person-centered statistical technique, LPA identifies unobserved latent profiles within a population based on responses across a set of continuous variables, enabling empirical examination of population heterogeneity beyond the assumption of a single uniform group [46].

The LPA was originally conducted in Mplus 8.3 and reproduced in R 4.2 using the mclust package [47], so that the analysis code could be made openly available. The six continuous variables representing the percentage of each student’s questions classified into the six Bloom’s Taxonomy categories [10,48] served as indicators. A series of models was estimated, starting with a two-profile model and progressively increasing the number of profiles, to identify the optimal solution. The equal-variance, no-covariance model (EEI parameterization) was used based on theoretical expectations that profiles would differ primarily in their mean levels rather than in the covariance structure among indicators. The reproduction in R yielded an identical profile composition, confirming the robustness of the solution across software.

Model selection was guided by multiple criteria: statistical fit indices including the Bayesian Information Criterion (BIC) and Integrated Complete-data Likelihood (ICL), where lower values indicate better model fit; conceptual distinctiveness and theoretical interpretability of the resulting profiles; entropy value, a measure of classification quality with values closer to 1.0indicating clearer separation between profiles; and average posterior probability (AvePP) with values above 0.70 indicating acceptable classification. Based on holistic evaluation of these criteria, the three-profile solution was identified as the most parsimonious and conceptually meaningful representation of the data, demonstrating good statistical fit (BIC = −4967.2, ICL = −4977.1), producing distinct and meaningful student profiles, and yielding an entropy value of 0.958 for high classification accuracy. The three-profile solution also showed robust AvePP values (0.974, 0.957, 0.954 for Profiles 1–3, respectively), all well above the 0.70 threshold.

#### Phase 3: GBTM – longitudinal trajectory patterns.

GBTM, a semi-parametric approach introduced by [49], was adopted to identify subgroups of students with homogeneous developmental trajectories of question types across conversation rounds. Unlike LPA, which focuses on static proportions of question types, GBTM empirically derives dynamic trajectories from longitudinal data without a priori assumptions about the number of subgroups or trajectory shape, complementing LPA by capturing temporal changes in cognitive engagement.

The GBTM was conducted using the Proc Traj procedure in SAS 9.4, with results reproduced and validated in R using custom EM-based estimation. The longitudinal data of coded question type values, reflecting cognitive engagement levels and collected per conversation round, served as the primary variables. Given the quantitative nature of the coded question types, the model was fitted based on a censored normal distribution. Participants with missing baseline data or fewer than 3 measurements were excluded to ensure data integrity.

**Fig 1:**
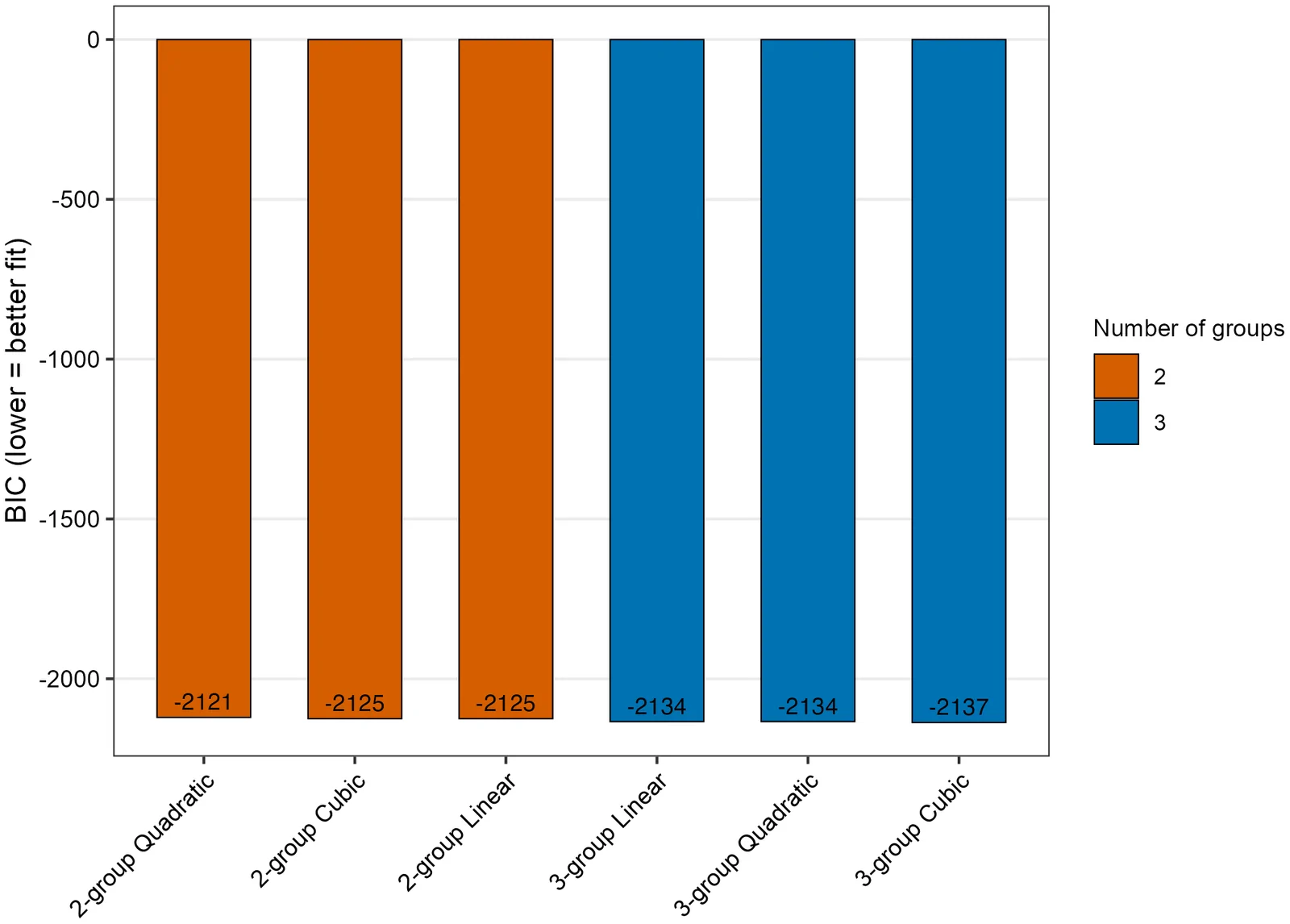
Sensitivity analysis of GBTM model specifications: BIC comparison across group numbers and polynomial orders.

A series of models was tested, varying the number of trajectory groups from 1 to 3 and trajectory functions from 1st-order linear, 2nd-order quadratic, to 3rd-order cubic. Model selection relied on a combination of statistical evaluation criteria and theoretical interpretability: average posterior probability (Avepp) with values greater than 0.70 indicating acceptable classification reliability; Odds of Correct Classification (OCC) with values greater than 5 denoting robust classification accuracy and values between 3 and 5 considered acceptable; BIC where lower values indicate better model fit, with △BIC representing the difference between the BIC of a complex model and the null model; relative entropy (Ej) with values closer to 1reflecting higher classification precision; and theoretical interpretability of the resulting groups. A sensitivity analysis comparing all two- and three-group models across polynomial orders was conducted to ensure that the selected model was not an artifact of a single specification (see Fig 1).

Among the tested specifications, the 2-group model with 3rd-order cubic trajectories (2Groups(33)) was selected as the optimal model. It exhibited a balanced sample distribution across groups, avoiding extreme subgroup sizes that limit interpretability, and the observed values and model-predicted values in the trajectory plot showed strong consistency. Statistically, the model achieved Avepp values well above the 0.7 threshold (Group 1: 82.1%, Group 2: 83.4%) and OCC values indicating acceptable to robust classification (Group 1: 4.27, Group 2: 5.42), with high congruence between observed and estimated group proportions. While the 3Groups(333) model yielded a marginally lower BIC (△BIC = −12.0), it contained an extremely small subgroup (n = 7, 6.6% of the sample) that was insufficiently large to support stable parameter estimation or meaningful interpretation. Following [49] and recommendations in the GBTM literature, models with subgroups comprising less than 5% of the sample should be treated with caution, and the 2-group solution was retained for its balance of statistical adequacy, subgroup size, and theoretical clarity.

#### Phase 4: Comparing subgroups/Trajectory groups on cognitive outcomes.

In the final phase, we examined whether different questioning patterns were associated with differences in students’ self-reported cognitive engagement. Specifically, both the three LPA-derived questioning profiles and the two GBTM-derived trajectory groups were compared on CT and CrT outcomes.

Two outcome sources were collected: (a) self-reported perceived CT and CrT engagement scores from the post-task questionnaire, and (b) objective performance scores derived from grading the submitted chat logs using CTAR [41] and CTR [42]. It is important to note that the CT measure was adapted from the Motivated Strategies for Learning Questionnaire [40], which assesses the frequency of learning strategy use during the task rather than measuring change in thinking ability. Accordingly, we frame these scores as task-specific self-reported CT/CrT strategy engagement rather than as measures of cognitive growth. For the main subgroup comparisons, self-reported CT and CrT scores were used, for three reasons. First, objective rubric scores primarily reflect the level of thinking evidenced in the final product, whereas the purpose of this study was to identify which questioning patterns are associated with differential self-reported engagement. Second, using rubric scores as the primary outcome risks criterion contamination because the objective ratings were based on the same chat logs from which questioning patterns were coded; students who produced more higher-order questions would, by design, be more likely to receive higher rubric scores, inflating associations between questioning patterns and “outcomes.” Third, a pre–post testing design was not well-suited to this setting because the task involved a single dialogue activity with a short time window, making pre–post changes difficult to interpret as meaningful development. Nevertheless, to ensure that self-reported outcomes were not detached from demonstrated performance, we conducted a Pearson correlation analysis, which showed a significant and strong positive association between subjective and objective scores (CT: *r* = .642, CrT: *r* = .726; both *p <* .001). We note, however, that this correlation should be interpreted with caution because both the objective ratings and the questioning codes were derived from the same dialogue transcripts, and shared source variance may inflate the observed association. In addition, objective scores were on non-comparable metrics (CTAR: 1–6; CTR: 0–12), further reducing interpretability for direct cross-construct comparisons in subgroup analyses.

For the LPA profiles, students were assigned to the profile with the highest posterior membership probability. Because normality assumptions were not met within profiles, Kruskal–Wallis H tests were used to compare perceived CT and CrT across the three profiles, followed by Bonferroni-adjusted post hoc pairwise comparisons where omnibus effects were significant. For the GBTM results, students were assigned to the trajectory group with the highest posterior probability, and independent-samples t tests were conducted to compare perceived CT and CrT between the two trajectory groups; Levene’s test assessed homogeneity of variance, and Cohen’s d was reported to quantify effect size.

### 3.4 Ethics

Ethical approval was obtained from the University Ethics Review Committee of the Chinese University of Hong Kong, Shenzhen in November 2024. The formal approval was documented through an officially signed and stamped ethics review resolution letter. Recruitment took place from December 1–31, 2024. Data collection was conducted from January 1 to June 1, 2025. No data were collected prior to ethics approval. All participants were adults; therefore, parental or guardian consent was not required, but individual informed consent was obtained from all participants. Written informed consent was obtained electronically via the online questionnaire. Participants reviewed an information/consent statement and indicated consent by selecting the “I agree to participate” option before proceeding; data were then analyzed in de-identified form.

## 4. Findings

### 4.1 Static questioning patterns: LPA

**Fig 2:**
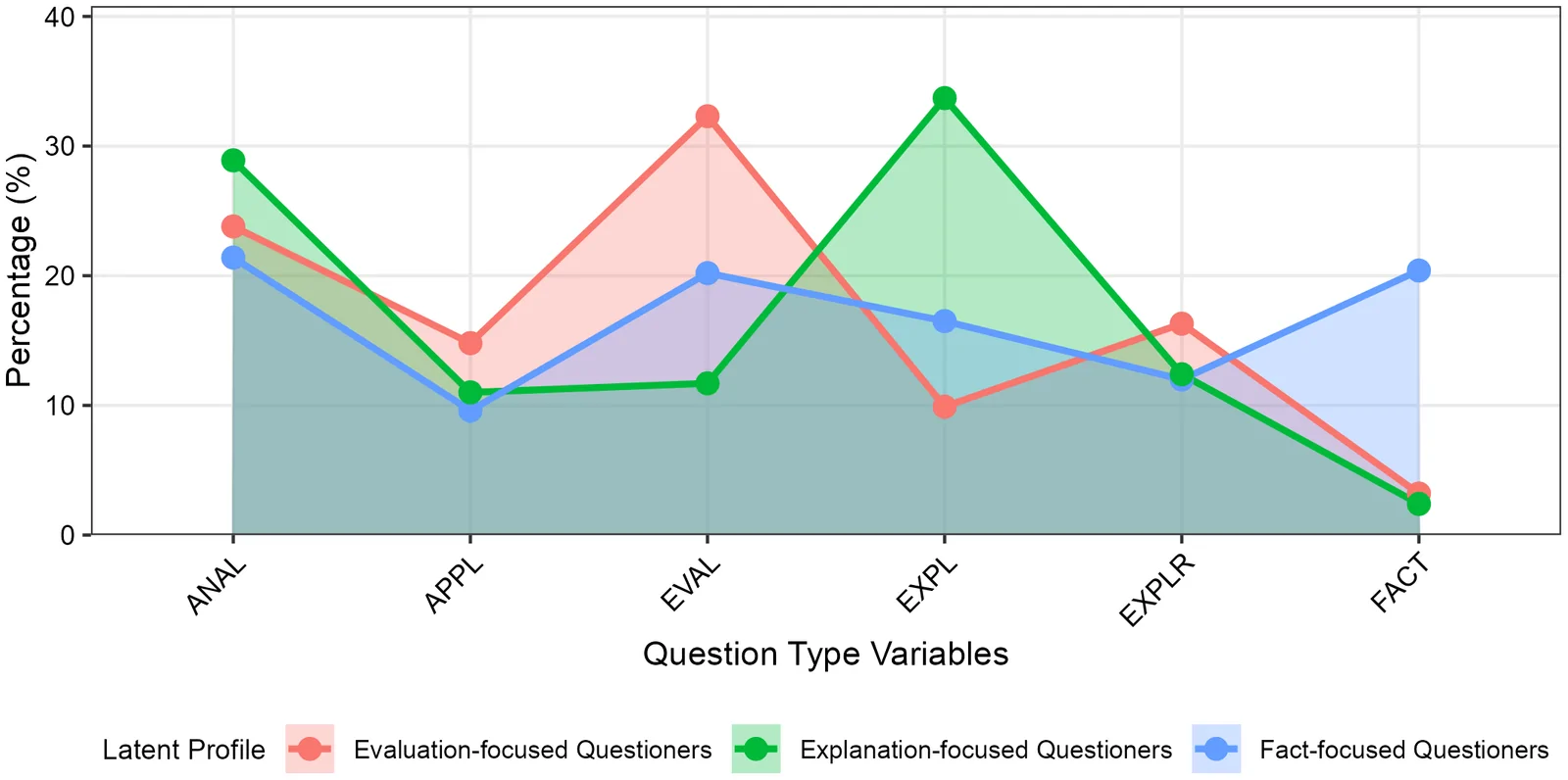
LPA results of students’ questioning patterns.

To address the first research question regarding students’ questioning patterns during dialogue with LLMs, an LPA was conducted on the coded chat log data. The analysis identified a three-profile solution as the optimal representation of the data, balancing statistical fit with conceptual clarity (see Fig 2). This model demonstrated high classification accuracy with an entropy value of 0.958.

The three distinct profiles, named according to their dominant questioning characteristics, included Fact-focused Questioners (n = 23), who were characterized by a significantly higher reliance on lower-order factual questions (21.0% of their total questions) compared to the other profiles, with engagement primarily aimed at information retrieval. Explanation-focused Questioners (n = 31) showed a pronounced emphasis on questions seeking more detailed understanding, with a high frequency of both explanatory (33.7%) and analytical (28.5%) questions, using the LLM to clarify concepts and explore relationships between ideas. Evaluation-focused Questioners (n = 52), the largest group, were distinguished by a major focus on higher-order evaluative questions (32.7%) and minimal use of factual questions (3.2%), engaging the LLM in more critical dialogue centered on judgments and assessing the value of information.

### 4.2 Longitudinal Trajectory Patterns: GBTM

Complementing static profiles, GBTM revealed two distinct longitudinal trajectories of cognitive engagement, addressing RQ2 by capturing how questioning patterns evolve over dialogue:

#### 4.2.1 Model fit and adequacy.

**Fig 3:**
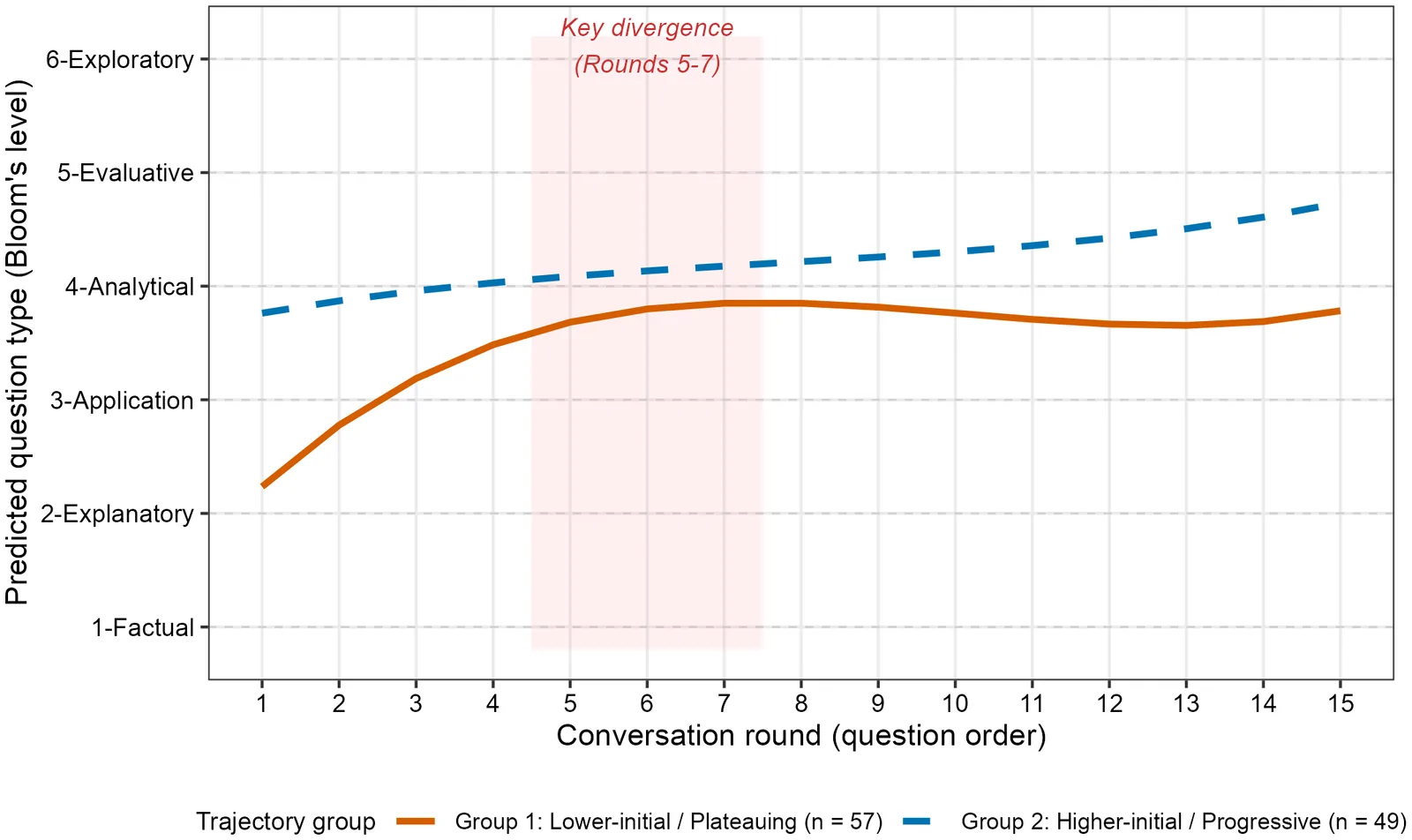
Trajectory plot of the optimal 2Groups(33) model.

The optimal 2Groups(33) model demonstrated robust reliability (see Tables 1–2, Fig 3). AvePP values (82.1% for Group 1, 83.4% for Group 2) exceeded the 0.70 threshold, OCC values (4.27 for Group 1, 5.42 for Group 2) indicated acceptable to robust classification, and observed group proportions (53.8% Group 1, 46.2% Group 2) closely matched estimated probabilities (51.8% Group 1, 48.2% Group 2).

**Table 1:** Fitting effect evaluation indicators of GBTM models.

Model	*AvePP%*	Proportions per class%	*π*_*j*_ *%*	*BIC*	*△BIC*	*OCC*	*E*_*j*_
**1Groups(2)**	100.0	106(100.0)	100.0	−2117.0			
**2Groups(11)**	78.3-84.0	22(20.8)-84(79.2)	28.9-71.1	−2125.0	−8.0	8.87-2.14	0.451
**2Groups(22)**	83.9-80.3	59(55.7)-47(44.3)	55.4-44.6	−2121.0	3.0	4.19-5.06	0.431
**2Groups(33)**	**82.1-83.4**	**57(53.8)-49(46.2)**	**51.8-48.2**	**−2125.0**	**−3.0**	**4.27-5.42**	**0.440**
**3Groups(111)**	79.2-63.2-79.5	6(5.7)-28(26.4)-72(67.9)	10.8-28.7-60.5	−2134.0	−9.0	31.41-4.26-2.52	0.459
**3Groups(222)**	81.9-78.8-75.2	58(54.7)-45(42.5)-3(2.8)	52.9-43.4-3.7	−2134.0	0.0	4.03-4.84-78.84	0.581
**3Groups(333)**	75.9-76.7-82.8	52(49.1)-7(6.6)-47(44.3)	45.8-7.7-46.5	−2137.0	−4.0	3.73-39.42-5.56	0.552

**Table 2:** Model adequacy diagnostics for optimal 2Groups(33) model.

Model	Group1	Group2
Number of students (%)	57(53.8%)	49(46.2%)
(a) Average posterior probability value (>0.70)	82.1%	83.4%
(b) Odds of correct classification (>5)	**4.27**	5.42
(c) Estimated group probability	51.8%	48.2%

#### 4.2.2 Trajectory characteristics.

Group 1 (n = 57; ŷ = 1.548135 + 0.765667x − 0.081039x² + 0.002662x³) started at a relatively low questioning level, with questioning dominated by Factual questions and some Explanatory questions in the early rounds. Their cognitive level increased rapidly across the first few rounds and, by approximately Round 5 to Round 7, their questioning largely stabilized around Levels 3–4, corresponding mainly to Application and Analytical questions. From that point onward, the trajectory shows a clear plateau with only modest fluctuation, indicating that this group tended to hover at mid-level cognitive engagement and found it difficult to progress consistently into higher-order questioning.

Group 2 (n = 49; ŷ = 3.629805 + 0.146072x − 0.013969x² + 0.000609x³) began at a higher questioning level, showing limited reliance on Factual questions and greater emphasis on Explanatory and Analytical questioning from the outset. Across the dialogue, Group 2 maintained a steadily upward trajectory rather than plateauing. In the later rounds, their questioning increasingly reflected higher-order engagement, moving toward Evaluative questions and, by the final rounds, showing evidence of reaching the most advanced levels of the framework, including Exploratory questions.

As shown in Fig 3, the key differences between the two trajectories involve initial questioning level, developmental pattern, and final level of engagement. Group 1 demonstrates rapid early growth followed by a sustained plateau around Application and Analytical questioning, whereas Group 2 sustains growth and is more likely to progress into Evaluative and Exploratory questioning toward the end of the dialogue.

### 4.3 Relationship Between Questioning Patterns/Trajectories and Self-Reported Cognitive Engagement

For the LPA profiles, Kruskal-Wallis H tests revealed statistically significant differences among profiles for both CrT (H(2) = 20.868, *p <* 0.001) and CT (H(2) = 11.550, *p <* 0.05). Post hoc pairwise comparisons with Bonferroni correction clarified these differences: Fact-focused Questioners reported significantly lower CrT (*M =* 4.30, *SD =* 0.78) and CT (*M =* 4.55, *SD =* 0.85) scores than both Evaluation-focused (CrT: *M =* 5.22, *SD =* 0.70; CT: *M =* 5.23, *SD =* 0.63) and Explanation-focused Questioners (CrT: *M =* 5.15, *SD =* 0.67; CT: *M =* 5.23, *SD =* 0.70). No statistically significant differences were found between Evaluation-focused and Explanation-focused groups in either CrT or CT scores, suggesting both higher-order questioning patterns were associated with similarly higher self-reported cognitive engagement (see Table 3).

**Table 3:** Descriptive and Kruskal-Wallis H test results for self-reported cognitive engagement across latent questioning profiles (N = 106).

Construct	Profile	N	Mean	SD	H (adj.)	*df*	*p*	Significant Pairwise Comparisons
**CrT**	**Fact-focused**	23	4.30	0.78	20.868	2	< .001	Fact < Eval; Fact < Expl
	**Evaluation-focused**	52	5.22	0.70				
	**Explanation-focused**	31	5.15	0.67				
**CT**	**Fact-focused**	23	4.55	0.85	11.550	2	.003	Fact < Eval; Fact < Expl
	**Evaluation-focused**	52	5.23	0.63				
	**Explanation-focused**	31	5.23	0.70				

*Note.* Fact: Fact-focused Questioners; Eval: Evaluation-focused Questioners; Expl: Explanation-focused Questioners. Adjusted significance values were calculated via Bonferroni correction. Effect size was not reported for non-significant comparisons.

For the GBTM trajectory groups, as shown in Table 4, independent samples t-tests showed the first group (lower initial engagement, rapid growth) had significantly lower CrT scores (*M =* 4.78, *SD =* 0.85) than the second group (higher initial engagement, stable growth; *M =* 5.26, *SD =* 0.64; *t*(104) = −3.22, *p <* 0.01) with a medium effect size (Cohen’s *d* = −0.63). The mean difference in CrT scores was −0.48 (95% CI: −0.77, −0.18), with Levene’s test indicating non-significantly different variances (*F* = 3.42, p > .05). For CT scores, the first group (*M =* 4.93, *SD =* 0.79) scored significantly lower than the second group (*M =* 5.26, *SD =* 0.70; *t*(104) = −2.26, *p <* 0.05) with a small-*t*o-medium effect size (Cohen’s *d* = −0.44). The mean difference in CT scores was −0.32 (95% CI: −0.61, −0.04), with Levene’s test confirming homogeneous variances (*F* = 1.07, *p* > 0.05).

**Table 4:** Descriptive and independent-samples t-test results for self-reported cognitive engagement across GBTM trajectory groups (N = 106).

Construct	Trajectory Group	N	Mean	SD	*t*(104)	*p*	Cohen’s d	95% CI
CrT	Group 1 (Plateauing)	57	4.78	0.85	−3.22	.002	−0.63	[-0.77, -0.18]
	Group 2 (Progressive)	49	5.26	0.64				
CT	Group 1 (Plateauing)	57	4.93	0.79	−2.26	.026	−0.44	[-0.61, -0.04]
	Group 2 (Progressive)	49	5.26	0.70				

*Note.* Group 1: Lower-initial questioning level with plateauing trajectory; Group 2: Higher-initial questioning level with progressive trajectory. Cohen’s d reflects the magnitude of difference (Group 1 − Group 2). Equal-variance t-test was used; Levene’s test confirmed non-significant variance differences for both CT (F = 1.07, p > .05) and CrT (F = 3.42, p > .05).

## 5. Discussion

This study examined how students’ questioning functions as an interaction mechanism in student–LLM philosophical dialogue and how different questioning patterns relate to self-reported CT and CrT engagement. Across both static and longitudinal analyses, the results point to a consistent pattern: questioning profiles and trajectories are associated with differential self-reported cognitive engagement, and self-reported CT engagement is associated with how learners use questioning to sustain inquiry beyond mid-level sensemaking. In particular, profile differences showed that students who relied heavily on factual questioning reported lower CT and CrT engagement scores, while trajectory differences suggested a key divergence around Rounds 5–7, when some students plateaued at application and analytical questioning rather than advancing toward evaluative and exploratory questioning.

### 5.1 Interpreting heterogeneity in student questioning (RQ1)

The three questioning profiles indicate that students enacted different inquiry orientations in student–LLM dialogue. This aligns with accounts that treat questioning as an epistemic process rather than a surface discourse feature [17,50–52]. In this view, questions can function either to obtain information or to probe meanings, assumptions, and implications. The Fact-focused profile reflects a stronger emphasis on information-oriented questioning, whereas the Explanation- and Evaluation-focused profiles reflect greater use of questions that support sensemaking and judgment. This profile-based heterogeneity is also consistent with classroom discourse research showing that participation structures can channel learners into answer-oriented routines or into more inquiry-driven talk [14–16]. Pedagogically, it also resonates with the argument that productive questioning is not simply a hierarchy of levels but a flexible practice that can connect subject matter with personal and broader social realities [13].

### 5.2 Understanding questioning trajectories as dynamic inquiry processes (RQ2)

The trajectory results suggest that questioning in student–LLM dialogue develops over time and can follow distinct developmental patterns. This supports prior work that conceptualizes questioning as a dynamic interaction process that emerges and evolves through ongoing interaction [36]. In inquiry settings, questions are not fixed; they can be refined, transformed, or replaced as learners encounter new information and negotiate tensions in understanding [36]. The two trajectories identified here capture such temporal divergence across conversation rounds, rather than treating questioning as a single aggregate tendency. This process perspective is compatible with research in science education that links higher-quality inquiry to questions that open investigation and require interpretation and reasoning, rather than only recognition or recall [22,23].

### 5.3 Explaining differences in self-reported CT engagement (RQ3)

RQ3 was examined through two sets of comparisons: differences in self-reported CT engagement across the LPA-derived questioning profiles and differences across the GBTM-derived trajectory groups. Together, these results clarify which students reported higher CT engagement scores and what patterns of questioning development characterized those differences in student–LLM dialogue.

#### 5.3.1 Why Fact-focused Questioners reported lower CT and CrT engagement scores?.

Fact-focused Questioners reported significantly lower self-reported engagement in both CT and CrT than the other two profiles. For CT, this profile is consistent with a greater reliance on fact-finding questioning, which tends to privilege confirmation and information retrieval over probing assumptions, warrants, and implications. In contrast, Ikuenobe [17] characterizes analytical questioning as the form of inquiry that more directly supports critical thinking because it requires examining meanings, reasons, and justifications. When student–LLM dialogue is dominated by fact-focused prompts, the interaction is less likely to elicit sustained evaluation and self-regulatory judgment, which are central to CT [53].

For CrT, the same profile difference can be interpreted in terms of the breadth and generativity of the inquiry space that students open through their questions. Fact-focused questioning is associated with a narrower dialogue space focused on convergent answer production, with comparatively fewer opportunities to reframe the problem, generate alternatives, or combine ideas in new ways. By contrast, Explanation-focused and Evaluation-focused Questioners more frequently asked questions that invite elaboration, comparison, and appraisal, which may create openings for further exploratory lines of inquiry. This interpretation is consistent with pedagogical accounts that emphasize higher-order questions as those that connect content with broader contexts and values and thereby support deeper consideration and generative thinking [13]. It also aligns with inquiry-oriented perspectives in which questions that open investigation and require interpretation are more likely to drive productive exploration beyond recall [22,23].

#### 5.3.2 Why plateauing around Rounds 5–7 distinguished lower- and higher-engagement students?.

Beyond static profiles, the trajectory results indicate that self-reported CT engagement also differed depending on whether students’ questioning development progressed beyond a mid-dialogue plateau. Around Rounds 5–7, some students’ questioning stabilized at application and analytical levels rather than advancing toward evaluative and exploratory questioning. One plausible interpretation is that this divergence reflects underlying epistemic orientations toward knowledge and inquiry. In fallibilist accounts, questioning is valued because beliefs are treated as revisable and must be continually evaluated and justified through inquiry [17–21]. However, as [17] argues, students may also approach learning as locating a correct answer and terminating inquiry once an answer is obtained, which limits deeper exploration of assumptions, implications, and alternatives [17]. Applied to student–LLM dialogue, plateauing at application and analytical questioning may signal that learners have reached a locally satisfactory explanation and shifted into answer-settling, whereas continued movement into evaluative and exploratory questioning may reflect a more open-ended stance that sustains judgment and alternative generation.

This interpretation is consistent with process-oriented perspectives that treat questioning as a dynamic interaction mechanism that can evolve as learners encounter tensions between ideas, evidence, and competing viewpoints [36,54,55]. In our data, learners who remained at application and analytical questioning likely engaged in meaningful sensemaking, but they did not consistently transition into the evaluative and exploratory questioning that more directly enacts criteria-based judgment and the testing of claims against alternatives, which are central components of CT [53]. Accordingly, students who plateaued around Rounds 5–7 reported lower CT engagement scores than those whose questioning continued to develop into evaluative and exploratory forms in later rounds.

### 5.4 Implications

For instructors and course designers, the results suggest that simply encouraging students to “use an LLM” is insufficient; support should focus on helping students shift from information-oriented prompting to evaluative and exploratory questioning, especially around the mid-dialogue phase where many students plateau. For students, the results highlight questioning as a controllable learning strategy: using the LLM as a partner for critique, justification, and alternative generation is associated with higher self-reported cognitive engagement than treating it as an answer provider. For educational technology developers and institutional adopters, the results point to concrete opportunities for interface and workflow support, such as embedding prompt scaffolds that cue evaluation and exploration, and providing analytics-based feedback that flags mid-dialogue stagnation and recommends next-step question moves.

### 5.5 Limitations

Several limitations should be noted. First, the single-course and single-university design limits both generalizability and causal inference; the observed associations may reflect pre-existing individual differences rather than effects of questioning. Second, the primary outcomes were self-reported strategy engagement collected after a single dialogue activity, not objective pre–post change scores; accordingly, the findings reflect associations with task-specific engagement rather than evidence of cognitive growth, and the correlation with objective scores (CT: r = .642; CrT: r = .726) should be interpreted with caution due to shared source variance from the same dialogue transcripts. Third, although the sample of N = 106 was adequate for model estimation, relatively small subgroups (e.g., n = 23 for the Fact-focused profile) limit the precision of parameter estimates, and both LPA profiles and GBTM trajectories should be treated as exploratory pending replication with larger samples. Fourth, although inter-rater reliability was established on a randomly selected 20% subset (κ = 0.704, substantial agreement) and subsequent coding followed a calibrated procedure with ongoing consultation on uncertain cases, reliability was not independently verified for the full corpus.

## 6. Conclusion

This study investigated which questioning patterns are associated with differential self-reported cognitive engagement in student–LLM philosophical dialogue by analyzing questioning as a key interaction mechanism. Using coded dialogue transcripts from an authentic course assignment, we identified three static questioning profiles and two longitudinal questioning trajectories. Students who relied more heavily on factual questioning reported lower self-reported CT and CrT engagement, whereas students who engaged more in explanatory and evaluative questioning reported higher engagement scores. Trajectory analysis further showed a meaningful mid-dialogue divergence: some students plateaued around Rounds 5–7 at application and analytical questioning, while others continued toward evaluative and exploratory questioning, and these patterns were associated with differential self-reported cognitive engagement.

Future research can extend this work by replicating the profile–trajectory patterns across disciplines and task types, incorporating direct measures of learners’ epistemic beliefs, employing pre–post designs with control groups, and experimentally testing whether targeted scaffolds introduced around the mid-dialogue plateau can help more students transition into evaluative and exploratory questioning.
